# Aortic valve calcium volume as measured by native versus contrast-enhanced computer tomography and the implications for the diagnosis of severe aortic stenosis in TAVR patients with low-gradient aortic stenosis

**DOI:** 10.1186/s43044-022-00311-8

**Published:** 2022-09-30

**Authors:** Mohammad El Garhy, Tamer Owais, Philipp Lauten

**Affiliations:** 1 Department of Cardiology, Heart Centre, Zentralklinik Bad Berka, Robert-Koch Allee 9, 99437 Bad Berka, Germany; 2 Department of Cardiology, Minia University, Minia, Egypt; 3 Department of Cardiac Surgery, University Hospital Augsburg, Augsburg, Germany; 4 Department of Cardiothoracic Surgery, Cairo University, Cairo, Egypt

**Keywords:** TAVR, Computed tomography, Calcium, Aortic valve, Low-gradient aortic stenosis

## Abstract

**Background:**

Most of TAVR centers evaluate the calcium score in contrast-enhanced (ce) CT. We compared in this study between different methodologies to measure calcium score. We studied also the difference between patients with low-gradient (LG) and high-gradient (HG) severe aortic stenosis (AS) as regard the burden of aortic valve calcium (AVC).

**Results:**

We measured the calcium volume and score using Agatston methodology in non-contrast (nc) CT and with modified and fixed 850 Hounsfield unit (HU) thresholds in ce CT. The calcium score and volume in ceCT using even with modified thresholds is significantly lower than the assessed score and volume in ncCT. The median (IQR) of calcium score in nc CT and in cc CT were 1288 AU (750–1815) versus 947 HU (384–2202). The median (IQR) of calcium volume in nc CT and in cc CT with modified thresholds were 701 mm^3^ (239–1632) versus 197 mm^3^ (139–532). Agatston score and calcium volume were lower in patients with LG AS than HG AS; 2069 AU (899–2477) versus 928AU (572–1284) and 1537 mm^3^ (644–1860) versus 286 mm^3^ (160–700), respectively. Only 20% of patients with LGAS had Agatston score higher than the previously supposed AVC score threshold for the diagnosis of severe AS (> 2000AU in men and > 1200 in women).

**Conclusions:**

The diagnosis of severe LGAS should not depend on a single parameter as calcium score. In these patients, calcium score should be measured in nc CT and not in ce CT.

## Background

Calcific aortic stenosis (AS) is often, although not solely, an age-related condition in which scarring and degeneration of the aortic valve (AV) promotes deposition of calcium within it [1]. Computer tomography (CT) is a well-established method for the quantification of aortic valve calcium (AVC). Most prior studies have used non-contrast (nc) CT to assess AVC as part of the evaluation of AS severity. The cutoff values for this purpose differed between males and females (≥ 2000 or ≥ 3000 for men and ≥ 1200 or ≥ 1600 for women [2, 3]. The CT protocol used in these studies was similar to that used by Agatston for calcium scoring of coronaries [4]. AVC not only can aid in determining the severity of AS, but also can predict prognosis in AS patients [2]. These cutoff values are only validated in patients with high-gradient AS (HG AS) [5]. Discordant AS would encompass classical low flow low gradient (cLFLG), paradoxical LFLG, normal flow LG, and also patients with mean pressure gradient (PG) > 40 mm Hg and aortic valve area index (AVAi) > 0.6 cm^2^. Clavel and colleagues have reported that only 50% of patients with discordant AS had higher values than the cutoff value. Patients with discordant AS also had a lower AVC burden [6], and this group of patients accounts for approximately 30% of TAVR patients. The PG across the AV is determined not only by the AVarea, but also independently by flow in the LVOT, AVC load, and systemic arterial compliance [5]. Veulemans et al. showed that CT can differentiate the severity of LG AS only in men [6]. This study evaluated the AVC in contrast-enhanced CT (ceCT) with fixed threshold of 600 Hounsfield (HU). This fixed threshold could underestimate the calcium [7], especially in patients with discordant AS and low AVC burden. Even a multicenter trial only included few patients with discordant AS (161 from nearly thousand patients) [8]. The evaluation of AVC using ce CT in patients with LG AS is therefore quite limited. Thus, we aimed to evaluate the AVC in both native and ce CT in patients with concordant AS and in patients with LGAS.

## Methods

### Study population

We included all patients (479 patients) whom were treated with TAVR in Zentralklinik Bad Berka between 11/2019 and 12/2020 for symptomatic severe aortic stenosis with effective orifice area < 1.0 cm^2^. We excluded patients with valve-in-valve TAVR (11 patients) and patients with normal flow low-gradient aortic stenosis (6 patients). Indications for TAVR, device type, and approach were based on the assessment of the heart team. We divided patients into two groups according to mean pressure gradient over the aortic valve, group I with mean PG ≥ 40 mmHg and group II with mean PG < 40 mmHg. All patients in LFLG group had a mean transvalvular gradient < 40 mm Hg, effective orifice area < 1.0 cm2, and stroke volume index < 35 ml/m^2^.

### Doppler echocardiography

The main methodolgy to confirm the diagnosis of the aortic stenosis was the transthoracic echocardiography. The maximum PG was measured in apical five chamber, apical long axis, right parasternal, and suprasternal views. The aortic valve area was measured according to continuity equation. A dobutamine examination was performed only in patients with LG AS and LVEF < 50%. LVEF was measured by the biplane Simpson method. Stroke volume was measured in the LV outflow tract and was indexed to body surface area. LG AS was included in this study only if they also had low flow, SVi < 35 ml/m^2^.

### Pre-procedure cardiac CT angiography

For all patients, we analyzed the MSCT images, which were performed as standard-of-care pre-TAVR. Patients were evaluated using a Siemens Somatom Definition Edge scanner (Siemens Medical Solutions) using collimation of 0.6 mm at a fixed pitch of 0.2 with an injection of 70 ml of iopamidol (Ultravist-370; Bayer Vital Pharma). A dedicated protocol was formulated, with kV and tube current modified according to the patient’s size. Image acquisition for the heart was performed with retrospective ECG gating. CT Digital Imaging and Communications in Medicine (DICOM) data were analyzed using Siemens syngo software, Syngo Via, for TAVR Planning. In patients with LG AS, the cardiac output was measured to assure that stroke volume index is ≤ 35 ml/m2. So all patients with LG AS in this study also have low flow status.

#### Measurement of calcium volume

**Fig. 1:**
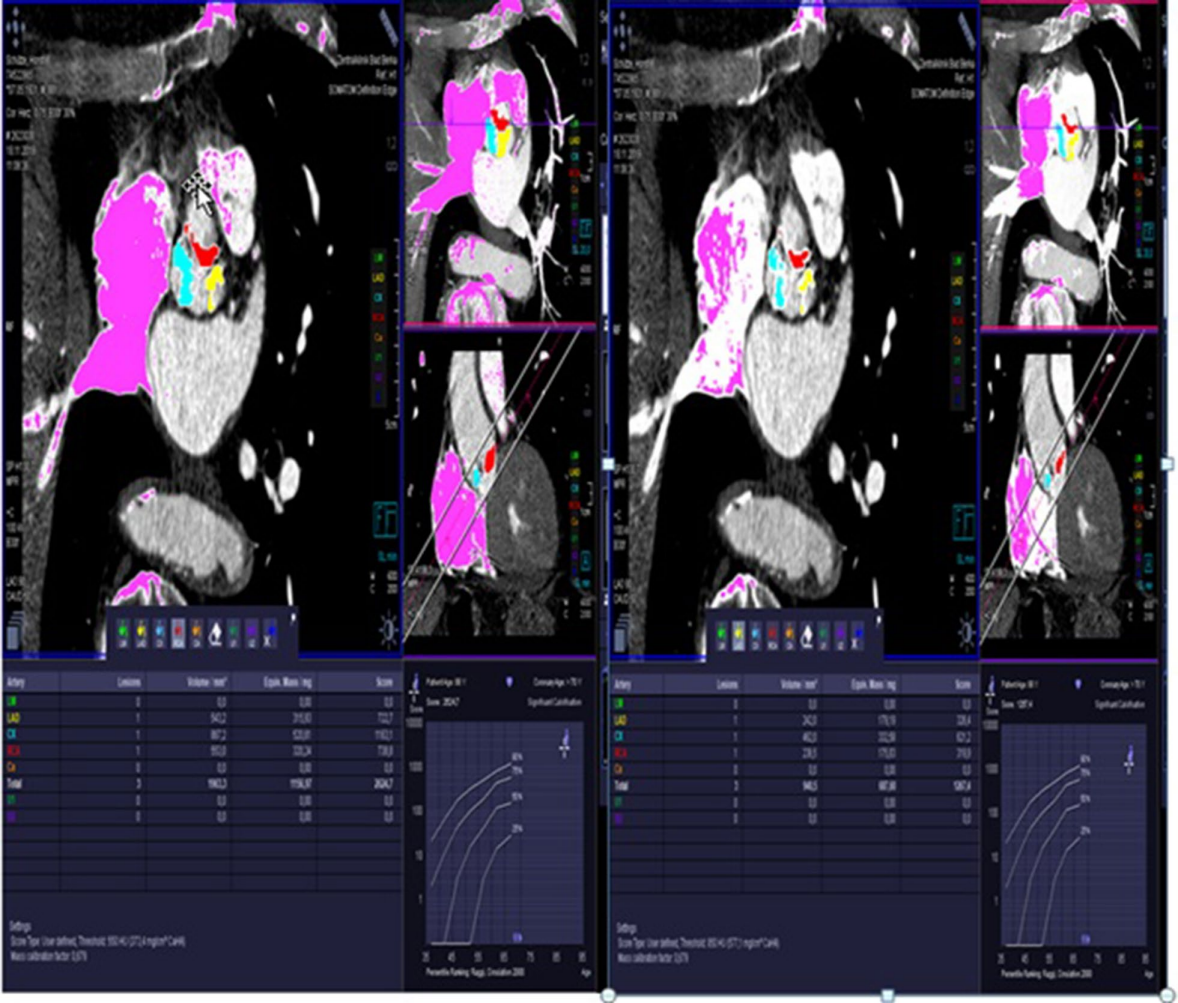
Calcium score and volume measurement use different HU threshold leads to change the score and volume significantly (to the right with 850 HU threshold and to the left with modifiable threshold)

The calcium score and volume of the aortic valve and each cusp were evaluated by the specialist (ME) using three different methodologies: (1) in nc CT imaging using a threshold of 130 Hounsfield (HU), (2) in ce CT scans using a modifiable threshold and a fixed threshold of 850 and 600. The modification of Hounsfield was used; thus, 100 HUs were added to the luminal attenuation HU [9], Fig. 1.

#### Assessment of radiation dose

**Fig. 2:**
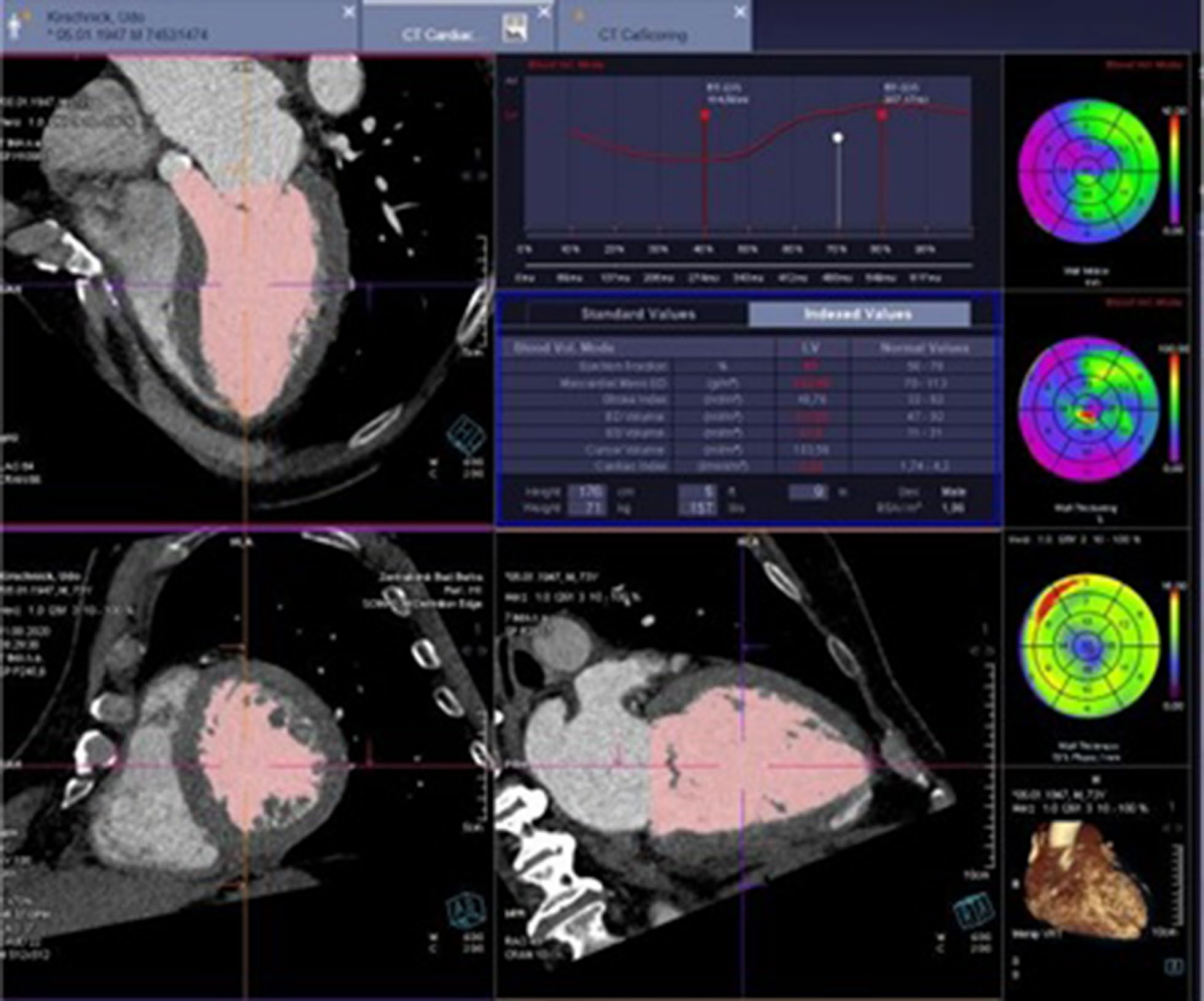
The use of the CT to measure the stroke volume especially in LG aortic stenosis

Radiation exposure was measured according to the methods previously described by Shnayien and colleagues. The dose-length product (DLP) was obtained from an automatically generated protocolthat was based upon the CT dose index (CTDI) and was measured in mGy * cm. The effective dose (*E*) was measured in mSv and was derived from the DLP as suggested by the European Guidelines on Quality Criteria for Computed Tomography. Thus, we used a conversion coefficient (*k*) of 0.017 and the following formula: *E* = *k* × DLP. The size-specific dose estimate (SSDE) is given in mGy, and was determined by multiplying conversion coefficients as a function of the sum of the lateral and anteroposterior dimensions with CTDI [10] (Fig. 2).

## Statistical analysis

Continuous variables were tested for normality of distribution by using the Shapiro–Wilk test. Normally distributed variables were expressed as mean ± standard deviation. For non-normally distributed variables, the median and inter-quartile range (IQR) were calculated and tested for statistical significance with the Mann–Whitney U test. Categorical variables were compared by Chi-square statistics. Statistical analyses were performed with SPSS (version 24.0; IBM Corporation, Armonk, NY). A two-sided *p* < 0.05 was considered statistically significant (Table 1).

**Table 1:** Preoperative patients’ characteristics

	HG AS 293 (61.2%)	LG AS 186 (38.8%)	Total 479	*p* value
Female *n* (%)	137 (46.8%)	82 (43.9%)	219 (45.6%)	0.5
Age median (IQR)	80 (77–83)	80 (78–83)	80 (77–83)	ns
BMI	27.6 (26–31)	31.2 (29.1–35.1)	29.1 (26.2–31.2)	0.04
EUROII median (SD)	26.6 (16.8)	28.2 (7.4)	27.1 (11)	0.07
Sts score	5.1 (4.8)	9.6 (14)	6.8 (8)	0.04
DM *n* (%)	133 (45.5%)	96 (51.3%)	229 (47.8%)	0.2
CLD *n* (%)	37 (12.6%)	36 (19.3%)	73 (15.2%)	0.06
CKD *n* (%)	69 (23.5%)	58 (31%)	127 (26.5%)	0.07
Stroke *n* (%)	15 (5.1%)	17 (9.1%)	32 (6.7%)	0.07
CABG *n* (%)	11 (3.8%)	15 (8%)	26 (5.4%)	0.04
Atrial fibrillation	84 (28.7%)	69 (36.9%)	153 (31.9%)	0.09
NYHA III/IV *n* (%)	178 (60.8%)	127 (67.8%)	304 (63.5%)	0.053

*BMI*, body mass index; *HTN*, hypertension; *CLD*, chronic lung disease; *CKD*, chronic kidney disease; *NYHA*, New York heart association

## Results

We included in this study 479 moderate to high-risk TAVR patients with mean STS mortality score from 6.8% and median age from 80 years (77–83). Forty-five percent (45.6%) from our patients were female and 38.8% had LG aortic stenosis. The median (IQR) of BMI was 29.1 (26.2–31.2) and of BSA was 1.91 (1.75–1.93). The calcium score and volume in ce CT using fixed 850 HU or modified thresholds is significantly lower than the assessed score and volume in ncCT. The median (IQR) of calcium score in nc CT and in cc CT with 850HU threshold and modified thresholds were 1288 AU (750–1815), 141 HU (84–351) (*p* value 0.001), 947 HU (384–2202) (*p* value 0.03), respectively. The median (IQR) of calcium volume in nc CT and in cc CT with 850HU threshold and modified thresholds were 701 mm^3^ (239–1632), 186 mm^3^ (114–426) (*p* value 0.02), 197 mm^3^ (139–532) (*p* value 0.02), respectively.

From 479 patients, 186 patients (38.8%) had LG AS. LG AS patients were more obese than patients with concordant AS; BMI 31.2 (29.1–35.1) versus 27.6 (26–31) and also presented more often with coronary artery disease (71.4% vs 40%). Atrial fibrillation was documented in 42% of the LG patients versus 30% in high-gradient patients (Table 2).

**Table 2:** Preoperative TTE and CT characteristics

	HG AS 293 (61.2%)	LG AS 186 (38.8%)	Total 479	*p* value
*Echocardiographic parameters*				
Max PG mmHg	71 (64–91)	48 (32–59)	63 (48.7–84.5)	< 0.05
Mean PG mmHg	46 (42–59)	32 (26–35)	41 (32.7–49.7)	< 0.05
AVA cm^2^	0.83 (0.7–0.9)	0.7 (0.7–0.8)	0.75 (0.7–0.9)	ns
RVSP mmHg	38 (12)	50 (15)	41 (13)	< 0.05
LVEF %	54 (14)	47 (11)	51.2 (13)	ns
More than mod. MR	69 (23.3)	45 (24.2)	114 (23.8)	ns
More than mod.TR	37 (12.6)	37 (19.9)	74 (15.4)	< 0.05
*CT parameters*				
Mean annulus D (mm)	25 mm (23–25.5)	23.5 mm (21.5–27)	23.5 (23–26)	ns
Max annulus D (mm)	26 (25–28)	26 (25–28)	26 (22–30)	ns
Min annulus D (mm)	23 (20–24)	20 (19–24)	21.5 (20–24)	< 0.05
LVOT (mm)	25 mm (23–26.7)	23 (20–20)	25 (22–26)	< 0.05
Eccentricity index	0.11 (0.1–0.2)	0.23 (0.19–0.27)	0.17 (0.1–0.21)	< 0.05
Annulus area (mm^2^)	504 (412–510)	388.5 (332–510)	458 (394–510)	ns
Annulus perimeter (mm)	78.5 (74–83)	72 (68.2–81)	76 (73–83)	ns
MS (mm)	3 (2.5–4.9)	5 (4–5.2)	3.7 (2.6–5.1)	ns
Aortic tilting angel	44 (43–45)	44 (44–48)	44 (43–48)	ns
Calc score (Agaston)	2069 (894–2477)	928 (572–1284)	1288 (750–1815)	< 0.05
Calc score (850 HU)	254 (158–583)	86 (11–107)	141 (89–351)	< 0.05
Calc score (modified HU)	1641(1292–1990)	392.5 (216–947)	947 (384–2202)	< 0.05
AVC V (mm^3^)	1537 mm^3^ (644–1860)	286 mm^3^ (160–700)	701 (239–1632)	< 0.05
AVC V (mm^3^) (850 HU)	101 (65–256)	51 (8–77)	186 (114–426)	< 0.05
AVC V (mm^3^) (modified HU)	266.5 (160–701)	137 (49–196)	197 (139–532)	< 0.05
Calc. LCC V (mm^3^) (modified HU)	320 (125–543)	113 (56–270)	150 mm^3^ (41–500)	< 0.05
Calc. RCC V (mm^3^) (modified HU)	600 (261–553)	94 (45–198)	289 mm^3^ (82–550)	< 0.05
Calc. NCC V (mm^3^) (modified HU)	732 (262–799)	208 (111–300)	300 mm^3^ (119–750)	< 0.05

*RBBB*, right bundle branch block; *LBBB*, left bundle branch block; *LAHB*, links anterior hemiblock; *PG*, pressure gradient; *AVA*, aortic valve area; *RVSP*, right ventricular systolic pressure; *LVEF*, left ventricular ejection fraction; *D*, diameter; *LVOT*, left ventricular outflow tract; *MS*, membranous septum; *Calc*, calcification; *DLZ*, device-landing zone; *LCC*, left coronary cusp; *NCC*, non-coronary cusp; *PPMI*, permanent pacemaker implantation; *RCC*, right coronary cusp; *AVC*, aortic valve calcification

LVEF was severely depressed (less than 30%) in 28.6% of LG patients. The mean PG in LG was 32 mmHg (25–35 mmHg) versus 46 mmHg (42–59 mmHg). Interestingly, LG patients were more symptomatic (NYHA ≥ III in 71.4% patients vs 42% of patients with high gradients). LG patients had also smaller dimensions in terms of their cardiac anatomy: annulus diameter 23.5 mm (21.5–27 mm) versus 25 mm (23–25.5 mm), LVOT diameter 23 mm (20–24 mm) versus 25 mm (23–26.7 mm). The annulus geometry of the LGAS group was more eccentric than in patients with concordant AS, with an eccentricity index of 0.23 (0.19–0.27) versus 0.11 (0.1–0.2). The LVOT of the LGAS patients was also more conical in shape. Agatston score and calcium volume were lower in patients with LG; 2069 AU (899–2477) versus 928AU (572–1284) and 1537 mm^3^ (644–1860) versus 286 mm^3^ (160–700), respectively. Only 20% of patients with LG had Agatston score higher than the previously supposed AVC score threshold for the diagnosis of severe AS (> 2000AU in men and > 1200 in women) (Table 3).

**Table 3:** Operative and postoperative data

	HG AS 293 (61.2%)	LG AS 186 (38.8%)	Total 479	*p* value
THV size (mm)	27 (26–29)	26 (23–29)	26.5 (23.5–29)	< 0.05
Type of THV				< 0.05
Edward Sapien	174 (59.3)	111 (39.1)	285 (59.4)	
Evolut	75 (25.6)	40 (21.4)	115 (24)	
AccurateNeo	23 (7.8)	28 (15)	51 (10.6)	
Lotus	21 (7.2)	8 (4.3)	29 (6)	
30-days mortality	5 (1.7%)	3 (1.6%)	8 (1.7%)	NS
VARC major bleeding	4 (1.4)	1 (0.5)	5 (1)	ns
Major vascular complication	8 (2.7)	8 (4.3)	16 (3.3)	ns
PPMI	49 (16.7)	32 (17.1)	68 (14.2)	ns
Acute kidney injury	36 (12.3)	32 (17.1)	68 (14.2)	ns
Non-disabling cerebral stroke	6 (2)	5 (2.7)	11 (2.3)	ns

*THV*, transcatheter heart valve, *VARC*, valve academic research consortium, *PPMI*, permanent pacemaker implantation

## Discussion

The main findings of this study were: firstly, that only 20% of severe AS patients with LG had a higher calcium score than the published cutoff recommended for the diagnosis of severe AS. Secondly, the calcium score and volume in ce CT using fixed 850 HU or even modified thresholds is significantly lower than the assessed score and volume using Agaston methodology in ncCT. Thirdly, Agatston score and calcium volume were lower in patients with LG than HG AS.

We also note that the mean AVC burden in this study was lower than in previously reported studies [6–8]. This could be explained that we included more patients with LGAS, this group of patients had lower calcium burden than HG AS. Other factors, which may affect AVC burden include hyperlipidemia, diabetes, chronic kidney disease, male sex, were comparable with the previous studies. We included high-risk patients with dyslipidemia (58%), diabetes (56.3%), and chronic kidney disease (17.6%). Thus, the risk profile in our study is comparable to that of prior studies, such as that of Aggarwal et al. In that study, the patients included had hyperlipidemia (67%), coronary artery disease (42%), and diabetes mellitus (24%). Likewise, the mean aortic annulus diameter (23.5 mm) and mean aortic annulus area (485.5 mm^2^) in our study were comparable to what Bittner et al. reported (24.2 mm and 462 mm^2^, respectively). We found that the distribution of calcification was uneven, being more prominent in the NCC and the RCC and less so in the LCC. This is consistent with Veulemans et al. who found that in AS of all severities and etiologies AS entities, the NCC was the most calcified. Cheng et al. also reported that the NCC had the most calcification and the LCC had the least calcification [11].

In previous CT studies of TAVR, the incidence of LG AS was between 33 and 56% [12], which is consistent with the 41.2% incidence we found. This high incidence of LG AS in TAVR patients highlights the importance of studying this entity. We demonstrate here that previously published CT-AVC thresholds are not applicable in LGAS. In keeping with our results, Pawade et al. found, in a multicenter study with 918 patients, that CT-AVC thresholds (women 1377 Agatston units, men 2062 Agatston units) were accurate only in patients with HG AS but not in patients with LG AS. Furthermore, Veulemans and colleagues found that the AVC load thresholds were only useful for differentiating between moderate and severe AS in both males and females when the AS was HG.

In the era of TAVR, this a common practice to measure the calcium volume in ce CT. In this study, the assessment of calcium volume using the 850 HU thresholds or modified threshold in ce CT underestimates the volume of calcium. So we recommend for the purpose of the diagnosis of the severity of aortic stenosis the use of the original Agaston methodology (150HU threshold) in native CT.

### Limitations

Some of the limitations of this study are that it is a single-center study and that its design is retrospective and observational. We need a large multicenter study to investigate the difference between calcium load in LG AS and HG AS.

## Conclusions

The diagnosis of severe LGAS should not depend on a single parameter as calcium score. In this patients calcium score should be measured in nc CT as the measurement of calcium score in contrast CT underestimates the calcium load significantly even with modifiable HU threshold.

## Data Availability

All data are available on request at the Department of Cardiology, Zentralklinik Bad Berka, Germany.

## Abbreviations

AVC: Aortic valve calcium
AU: Arbitrary unit
AS: Aortic stenosis
CT: Computed tomography
CE: Contrast enhanced
HU: Hounsfield units
HG: High gradient
ID: Implantation depth
LVOT: Left ventricle outflow track
LG: Low gradient
NPV: Negative predictive value
NCC: Non-coronary cusp
NC: Non-contrast
PPV: Positive predictive value
ROC: Receiver-operating characteristic
RCC: Right coronary cusp
TAVR: Transcatheter aortic valve replacement
